# ^68^Ga-labelled desferrioxamine-B for bacterial infection imaging

**DOI:** 10.1007/s00259-020-04948-y

**Published:** 2020-07-30

**Authors:** Milos Petrik, Eva Umlaufova, Vladislav Raclavsky, Andrea Palyzova, Vladimir Havlicek, Joachim Pfister, Christian Mair, Zbynek Novy, Miroslav Popper, Marian Hajduch, Clemens Decristoforo

**Affiliations:** 1 Institute of Molecular and Translational Medicine, Faculty of Medicine and Dentistry, Palacky University, CZ-77900 Olomouc, Czech Republic; 2 Department of Microbiology, Faculty of Medicine and Dentistry, Palacky University and University Hospital, Olomouc, Czech Republic; 3 Institute of Microbiology of the Czech Academy of Sciences v.v.i., Prague, Czech Republic; 4 Department of Analytical Chemistry, Faculty of Science, Palacky University, Olomouc, Czech Republic; 5 Department of Nuclear Medicine, Medical University Innsbruck, Anichstrasse 5, A-6020 Innsbruck, Austria

**Keywords:** Desferrioxamine-B, Gallium-68, PET, Infection, Imaging

## Abstract

**Purpose:**

With the increase of especially hospital-acquired infections, timely and accurate diagnosis of bacterial infections is crucial for effective patient care. Molecular imaging has the potential for specific and sensitive detection of infections. Siderophores are iron-specific chelators recognized by specific bacterial transporters, representing one of few fundamental differences between bacterial and mammalian cells. Replacing iron by gallium-68 without loss of bioactivity is possible allowing molecular imaging by positron emission tomography (PET). Here, we report on the preclinical evaluation of the clinically used siderophore, desferrioxamine-B (Desferal®, DFO-B), radiolabelled with ^68^Ga for imaging of bacterial infections.

**Methods:**

In vitro characterization of [^68^Ga]Ga-DFO-B included partition coefficient, protein binding and stability determination. Specific uptake of [^68^Ga]Ga-DFO-B was tested in vitro in different microbial cultures. In vivo biodistribution was studied in healthy mice and dosimetric estimation for human setting performed. PET/CT imaging was carried out in animal infection models, representing the most common pathogens.

**Results:**

DFO-B was labelled with ^68^Ga with high radiochemical purity and displayed hydrophilic properties, low protein binding and high stability in human serum and PBS. The high in vitro uptake of [^68^Ga]Ga-DFO-B in selected strains of *Pseudomonas aeruginosa*, *Staphylococcus aureus* and *Streptococcus agalactiae* could be blocked with an excess of iron-DFO-B. [^68^Ga]Ga-DFO-B showed rapid renal excretion and minimal retention in blood and other organs in healthy mice. Estimated human absorbed dose was 0.02 mSv/MBq. PET/CT images of animal infection models displayed high and specific accumulation of [^68^Ga]Ga-DFO-B in both *P. aeruginosa* and *S. aureus* infections with excellent image contrast. No uptake was found in sterile inflammation, heat-inactivated *P. aeruginosa* or *S. aureus* and *Escherichia coli* lacking DFO-B transporters.

**Conclusion:**

DFO-B can be easily radiolabelled with ^68^Ga and displayed suitable in vitro characteristics and excellent pharmacokinetics in mice. The high and specific uptake of [^68^Ga]Ga-DFO-B by *P. aeruginosa* and *S. aureus* was confirmed both in vitro and in vivo, proving the potential of [^68^Ga]Ga-DFO-B for specific imaging of bacterial infections. As DFO-B is used in clinic for many years and the estimated radiation dose is lower than for other ^68^Ga-labelled radiopharmaceuticals, we believe that [^68^Ga]Ga-DFO-B has a great potential for clinical translation.

**Electronic supplementary material:**

The online version of this article (10.1007/s00259-020-04948-y) contains supplementary material, which is available to authorized users.

## Introduction

Bacterial infections remain one of the leading cause of death globally [1]. Currently, hospital-acquired infections (HAIs) are among the most important public health issues, associated with high mortality and dramatic impact on healthcare costs [2, 3]. Increasing use of invasive techniques, immunosuppressive and cancer therapies in combination with increasing rates of drug-resistant pathogens remain a major challenge in controlling HAIs [1, 4]. The most common antibiotic-resistant bacteria being the leading cause of HAIs include *Enterococcus faecium*, *S. aureus*, *Klebsiella pneumoniae*, *Acinetobacter baumannii*, *P. aeruginosa* and *Enterobacter* species [5, 6], identified as the so-called “ESKAPE” microorganisms causing significant mortality and being a growing threat to public health [6–8].

The timely and accurate detection and localization of the causative agent responsible for an infection is crucial to avoid unnecessary antibiotic use and to focus appropriate, potentially life-saving therapy. Positive bacterial culture from the suspected sites (tissue and/or blood) is the current gold standard in the diagnosis of specific bacterial infection [9]. This method suffers from many limitations: (a) it can be invasive, (b) it is unable to determine extent of infection dissemination, (c) it lacks sensitivity often resulting in negative results, (d) is often only identified when it reached a systemic stage and (e) the time it takes to obtain results can often result in complications including fatality.

Molecular imaging provides the potential for specific, sensitive and location-specific imaging of infections, in particular based on radiotracers for SPECT and PET [10–16]. A number of non-specific radiotracers are applied clinically for imaging of infections [13–15]. These probes target predominantly secondary effects of infection, such as increased blood flow and vascular permeability, activated endothelial cells or polymorphonuclear cell migration.

Several attempts have been made to develop more specific radiotracers for microbial infections [1, 13–16]. An interesting group of molecules are radiolabelled siderophores [17], involving an active transport mechanism not existing in human cells that is highly activated during infection. Siderophores are low molecular mass iron chelators produced by nearly all microorganisms and some plants for iron acquisition and storage [18, 19]. Iron as a cofactor in basic metabolic pathways is essential to both pathogenic microorganisms and their hosts [20]. Given the absolute requirement for iron by virtually all human cellular pathogens, an important facet of the innate immune system is to limit iron availability to invading microbes in a process termed nutritional immunity [21] and having infections being described as “battle for iron” between host and pathogen [22].

There is no isotope of iron with suitable properties for nuclear imaging in terms of half-life and photon emission; however, Ga^3+^ is an isosteric diamagnetic substitute for Fe^3+^ and has been used extensively to characterize siderophore complexes [23]. In the past decade, interest in the isotope ^68^Ga, a positron emitter, has increased tremendously with the establishment of PET in clinic [24, 25]. ^68^Ga can be obtained from a ^68^Ge/^68^Ga generator and with a half-life of 68 min exhibits a very low radiation burden to the patient.

Here, we report on the possibility of repurposing of Desferal® (desferrioxamine-B, DFO-B) for diagnostic imaging of bacterial infections. DFO-B is a drug registered on the market for decades, containing as an active substance desferrioxamine-B, a hydroxamate siderophore produced by *Streptomyces pilosus*. DFO-B is clinically approved and effective for long-term metal chelation therapy in beta-thalassemia, sideroblastic anaemia, auto-immune haemolytic anaemia and other iron or aluminium overload cases. In this study, we have radiolabelled DFO-B with ^68^Ga and preclinically evaluated its potential for imaging of bacterial infections by means of PET.

## Materials and methods

### Chemicals

All reagents were purchased from commercial sources as reagent grade and used without further purification unless otherwise stated. Desferal® (deferoxamine mesylate for injection USP; MW = 656.8 g/mol) was purchased from Novartis (Basel, Switzerland). ^68^GaCl_3_ was eluted from a ^68^Ge/^68^Ga-generator (Eckert & Ziegler Eurotope GmbH, Berlin, Germany) with 0.1 N HCl using the fractionated elution approach.

### Radiolabelling and quality control

Radiolabelling of DFO-B with ^68^Ga was performed as follows: 20 μg of DFO-B dissolved in water (1 μg/μl) were mixed with 30 μl of sodium acetate (155 mg/ml in water) and 300 μl of ^68^Ge/^68^Ga-generator eluate (20–60 MBq of ^68^GaCl_3_). The reaction mixture (pH = 3–4) was incubated at 90 °C for l5 min. After the reaction, 100 μl of sodium acetate were added to increase the pH to 5–6. Radiochemical purity (RCP) of [^68^Ga]Ga-DFO-B was analysed by reversed-phase high-performance liquid chromatography (RP-HPLC) or using instant thin-layer chromatography on silica gel–impregnated glass fibres (ITLC-SG).

RP-HPLC analysis was performed with the following instrumentation: UltiMate 3000 RS UHPLC pump, UltiMate 3000 autosampler, Ultimate 3000 column compartment (25 °C oven temperature), UltiMate 3000 variable wavelength detector (Dionex, Germering, Germany; UV detection at λ = 220 nm), GABI Star radiometric detector (Raytest GmbH, Straubenhardt, Germany), ACE 3 C18; 150 × 3 mm, 3 μm column (Advanced Chromatography Technologies Ltd., Aberdeen, UK) with acetonitrile (ACN)/H_2_O/0.1% trifluoroacetic acid (TFA) as mobile phase; flow rate of 0.6 ml/min; gradient 0.0–2.0 min 8% ACN, 2.0–12.0 min 8–18% ACN, 12.0–15.0 min 50% ACN.

Radiolabelling efficiency and RCP were additionally analysed by ITLC-SG with a 1:1 (v/v) mixture of 1 M aqueous ammonium acetate and methanol as a mobile phase (pH = 7). The ITLC-SG strips (Varian, Lake Forest, CA, USA) were scanned using a Cyclone Plus Storage Phosphor System (PerkinElmer, Waltham, MA, USA).

### In vitro characterization

For the in vitro characterization of [^68^Ga]Ga-DFO-B, partition coefficient, protein binding values and stability in various media were examined. Partition coefficient (Log *P*) was determined by measuring the distribution of [^68^Ga]Ga-DFO-B between octanol and phosphate buffered saline pH = 7.4 (PBS). For this purpose, [^68^Ga]Ga-DFO-B was dissolved with PBS to 1 ml (~ 30 μM). Aliquots of 50 μl were added to 450 μl PBS and 500 μl octanol, and the mixture was vigorously vortexed for 20 min (at 1500 rpm) followed by centrifugation (2 min at 2000*g*). Hereafter, 50 μl of each phase were collected and measured in an automatic gamma counter 2480 Wizard^2^ (PerkinElmer, Waltham, MA, USA). Log *P* value was calculated using Microsoft Office Excel 2010 (mean of *n* = 6).

Protein binding studies were performed by incubating [^68^Ga]Ga-DFO-B in human serum and in PBS as a control at 37 °C up to 120 min. [^68^Ga]Ga-DFO-B was dissolved with PBS to 1 ml (~ 30 μM) and 50 μl of that solution was added to 450 μl human serum or 450 μl PBS. After 30-, 60- and 120-min incubation at 37 °C, aliquots of 25 μl were analysed by size exclusion chromatography using MicroSpin™ G-50 columns (Sephadex G-50, GE Healthcare, Buckinghamshire, UK) according to the manufacturer’s protocol. Protein binding of [^68^Ga]Ga-DFO-B was determined by measuring the activity distributed between the column (non-protein-bound) and the eluate (protein-bound) using a gamma counter.

The in vitro stability of [^68^Ga]Ga-DFO-B was tested in human serum, PBS, 6 mM diethylenetriaminepentaacetic acid (DTPA) and 0.1 M FeCl_3_ solution. Radiolabelled DFO-B was directly mixed with human serum and PBS in 1:10 ratio, while with DTPA and FeCl_3_ solutions in 1:1 ratio. Thus, prepared reaction mixtures were incubated for 30, 60 and 120 min, respectively, at 37 °C. After incubation, human serum samples were precipitated with acetonitrile and centrifuged (3 min, 2000*g*). The supernatant was analysed by RP-HPLC and/or ITLC-SG. Samples containing PBS, DTPA and FeCl_3_ were analysed directly. The stability is reported as radiochemical purity (% RCP) of [^68^Ga]Ga-DFO-B (*n* = 3).

### Microbial cultures and growth conditions

A part of the strains of *P. aeruginosa*, *E. coli* and *S. aureus* were obtained from the American Type Culture Collection (ATCC, Manassas, VA, USA), the National Collection of Type Cultures (NCTC, Salisbury, UK) and the Czech Collection of Microorganisms (CCM, Brno, Czech Republic). All other strains were clinical isolates from the Department of Microbiology, Faculty of Medicine and Dentistry, Palacky University and University Hospital Olomouc. Microbial strains used in the study are summarized in Supplementary Table S1. Microorganisms were maintained as frozen glycerol stock cultures at − 80 °C. One vial of the frozen stock culture of each strain was used as inoculum and ATTC or NCTC strain cultures were grown in a Luria-Bertani broth (LB; 1% tryptone, 0.5% yeast extract, 1% sodium chloride, pH = 7), while CCM strains and clinical isolates cultures were grown in Mueller Hinton broth. All the cultures were incubated at 28 °C for 24–48 h with shaking (200 rpm). To prepare solid media for determination of the number of colony-forming units (CFU) or a short-term strains maintenance, 22 g/l of agar were added to media.

### In vitro uptake and competition assays

Uptake assays were performed as previously published [26] in microbial cultures listed in Supplementary Table S1. Briefly, 500 μl of microbial cultures were incubated in Eppendorf tubes in triplicates with [^68^Ga]Ga-DFO-B (final concentration approximately 200 nM) at 37 °C for 45 min. The uptake was interrupted by 5-min centrifugation at 21000*g*. The supernatant was removed and the sediment was disturbed by 500 μl of ice-cold Tris buffer and subsequent whirling. The same procedure was repeated twice. After the last centrifugation and supernatant removal, Eppendorf tubes containing the microbial sediment were weighed and measured in a gamma counter. Results were expressed as percentage of applied dose per gram of microbial culture (%AD/g).

Competition assays were performed in the same way with a slight modification. Microbial cultures were pre-incubated with a high excess of iron-DFO-B (15 mM) for 20 min. The uptake value of [^68^Ga]Ga-DFO-B into bacteria was determined after another 45 min of incubation at 37 °C and the procedure described above.

### Animal experiments

Animal experiments were performed in female 8–10-week-old Balb/c mice and female 2–3-months-old Lewis rats (Envigo, Horst, The Netherlands). The animals were acclimatized to laboratory conditions for 1 week prior to experimental use and housed under standard laboratory conditions on sawdust in individually ventilated cages with free access to animal chow and water. During the experiments, general health and body weight of the animals were monitored. The number of animals was reduced as much as possible (generally *n* = 3 per group and time point) for all in vivo experiments. The introduction of bacterial infection into animals, [^68^Ga]Ga-DFO-B injection and small animal imaging was carried out under 2% isoflurane anaesthesia (FORANE, Abbott Laboratories, Abbott Park, IL, USA) to minimize animal suffering and to prevent animal motion.

### Ex vivo biodistribution

To evaluate pharmacokinetics and biodistribution of [^68^Ga]Ga-DFO-B in healthy animals, a group of three Balb/c mice per time point were retro-orbitally (r.o.) injected with [^68^Ga]Ga-DFO-B (1–2 MBq/mouse, 2 μg DFO-B). Animals were sacrificed by cervical dislocation at 10-, 30-, 60- and 90-min post-injection (p.i.). Organs and tissues of interest (blood, spleen, pancreas, stomach, intestines, kidneys, liver, heart, lung, muscle and bone) were collected, weighed and measured in a gamma counter. Results were expressed as percentage of injected dose per gram organ (%ID/g). Similarly, ex vivo biodistribution studies were performed also for murine *S. aureus* myositis model as well as intratracheally *P. aeruginosa* infected and non-infected rats. Mice and rats injected with [^68^Ga]Ga-DFO-B 5 h after inoculation were killed by exsanguination 45-min p.i. and collected organs were treated as described above.

### Dosimetric calculations

Dosimetric calculations were based on the biodistribution of [^68^Ga]Ga-DFO-B in healthy mice from 10- to 90-min p.i.. Clearance kinetics in each organ was determined by exponential curve fitting. The time-integrated activity concentration (TIAC) was obtained by integration of this exponential curve folded with the decay curve of ^68^Ga. Extrapolation of biodistribution uptake data in mice to humans was performed as described in [27]. The extrapolated human source organ residence times were used as input in the Olinda/EXM dosimetry software to calculate the absorbed doses per administered activity in humans [28].

### Animal imaging

PET/CT animal images were acquired with an Albira PET/SPECT/CT small animal imaging system (Bruker Biospin Corporation, Woodbridge, CT, USA). Animals were r.o. injected with [^68^Ga]Ga-DFO-B in a dose of 4–6 MBq corresponding to ~ 4 μg of DFO-B per animal. Anaesthetized animals were placed in a prone position in the Albira system before the start of imaging. Static PET/CT images were acquired 30–40 min starting 30 and 90 min after injection for normal biodistribution studies and 45-min p.i. for infection models imaging. A 10-min PET scan (axial FOV 148 mm) was performed, followed by a double (for mice) or triple (for rats) CT scan (axial FOV 110 or 160 mm, 45 kVp, 400 μA, at 400 projections). Scans were reconstructed with the Albira software (Bruker Biospin Corporation, Woodbridge, CT, USA) using the maximum likelihood expectation maximization (MLEM) and filtered backprojection (FBP) algorithms. After reconstruction, acquired data was viewed and analysed with PMOD software (PMOD Technologies Ltd., Zurich, Switzerland). Three-dimensional volume rendered images were obtained using VolView software (Kitware, Clifton Park, NY, USA).

### Animal infection models

In vivo uptake of [^68^Ga]Ga-DFO-B was studied in acute murine myositis and respiratory rat models. Murine myositis models were established by intramuscular (i.m.) injection of different strains of bacteria (live or heat-killed) or with turpentine oil to induce sterile inflammation. Mice were inoculated with 5 × 10^7^ (for *P. aeruginosa*) or 5 × 10^8^ (for *S. aureus*) CFUs of live bacteria in the left hind leg and with a tenfold higher burden of heat-killed bacteria or turpentine oil or 5 × 10^8^ (for *E. coli*) CFUs of live bacteria in the right hind leg. The microbial infections were allowed to develop for 5 h and sterile inflammation for 24 h. Animals were subsequently injected with [^68^Ga]Ga-DFO-B and imaged by means of PET/CT. For in vivo specificity challenge, the infection was induced in the left hind muscle with live *P. aeruginosa* ATCC 15692 or *S. aureus* CCM 5971 and in the right hind muscle with heat-killed (90 °C for 30 min) *P. aeruginosa* or *S. aureus*, or *E. coli* ATCC 10536 or turpentine oil.

Acute respiratory rat model was established as described previously [26]. Rats were infected intratracheally with *P. aeruginosa* ATCC 15692 under inhalation anaesthesia. *P. aeruginosa* (10^8^ CFU/dose in 100 μl) was administered using TELE PACK VET X LED system equipped with a flexible endoscope (Karl Storz GmbH & Co. KG, Tuttlingen, Germany). Five hours after inoculation, rats underwent ex vivo biodistribution or PET/CT imaging studies.

### Statistical and data analysis

All statistical analysis was performed using Microsoft Office Excel 2010. Data were analysed using an unpaired two-tailed Student’s *t* test. All graphs are depicted with error bars corresponding to the standard deviation. Other data including in vitro characterization of [^68^Ga]Ga-DFO-B are also reported as mean ± standard deviation.

## Results

### ^68^Ga-labelling and in vitro characteristics of [^68^Ga]Ga-DFO-B

**Fig. 1:**
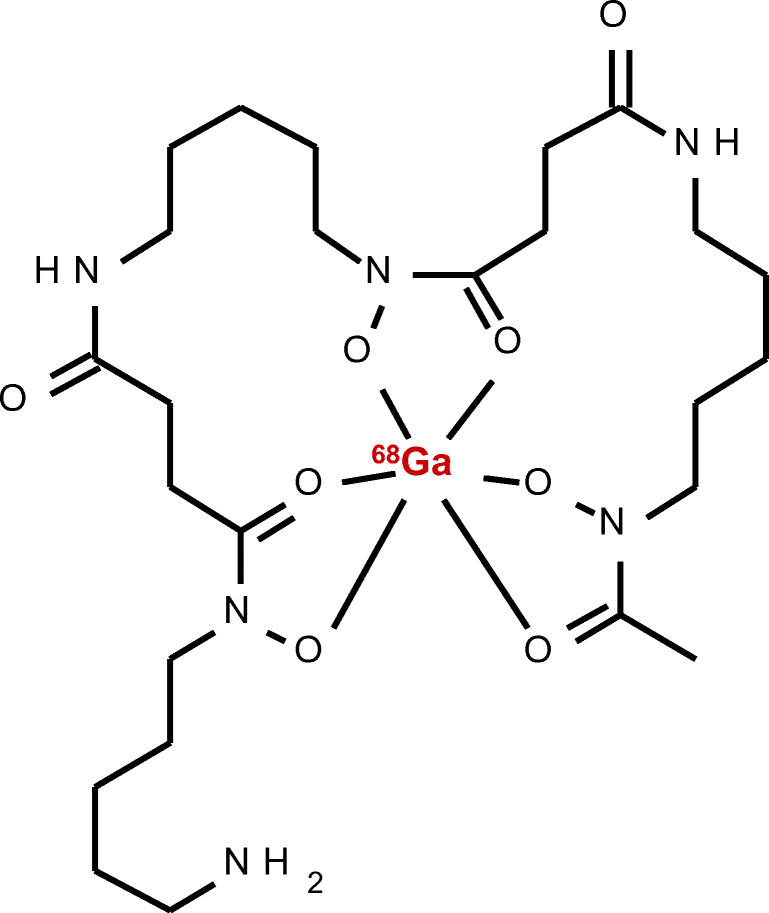
The chemical structure of [^68^Ga]Ga-DFO-B

DFO-B was radiolabelled with ^68^Ga (Fig. 1) with the molar activity up to 2 GBq/μmol and radiochemical purity > 95% confirmed by both RP-HPLC and ITLC-SG methods. In vitro assays showed hydrophilic properties of [^68^Ga]Ga-DFO-B (log *P* = −3.31 ± 0.19) with rather low protein binding tendency (~ 20% up to 120-min incubation) and high stability in human serum and PBS (> 98% for both). In other tested media, [^68^Ga]Ga-DFO-B displayed certain instability in DTPA solution (~ 30% after 120 min of incubation) and rapid in vitro instability in highly concentrated FeCl_3_ solution. The analytical and in vitro characteristics data of [^68^Ga]Ga-DFO-B are summarized in Supplementary Fig. S1 and Table 1.

**Table 1:** In vitro characterization of [^68^Ga]Ga-DFO-B. Log *P*, protein binding (expressed as percentage of protein-bound activity of the total activity used) and stability in human serum, PBS, 0.1 M FeCl_3_ and 6 mM DTPA

Log P (*n* = 6)	Incubation time (min)	Protein binding (%) (*n* = 3)	Stability in human serum (%) (*n* = 3)	Stability in PBS (%) (*n* = 3)	Stability in iron solution (%) (*n* = 3)	Stability in DTPA solution (%) (*n* = 3)
− 3.31 ± 0.19	30	19.9 ± 2.96	98.3 ± 0.83	99.5 ± 0.21	0.37 ± 0.12	94.1 ± 0.31
	60	21.8 ± 1.83	98.9 ± 0.33	99.5 ± 0.05	0.13 ± 0.05	85.3 ± 0.66
	120	22.5 ± 1.36	98.4 ± 0.46	98.0 ± 1.22	0.20 ± 0.01	71.2 ± 1.07

### In vitro uptake of [^68^Ga]Ga-DFO-B in different bacterial cultures

**Fig. 2:**
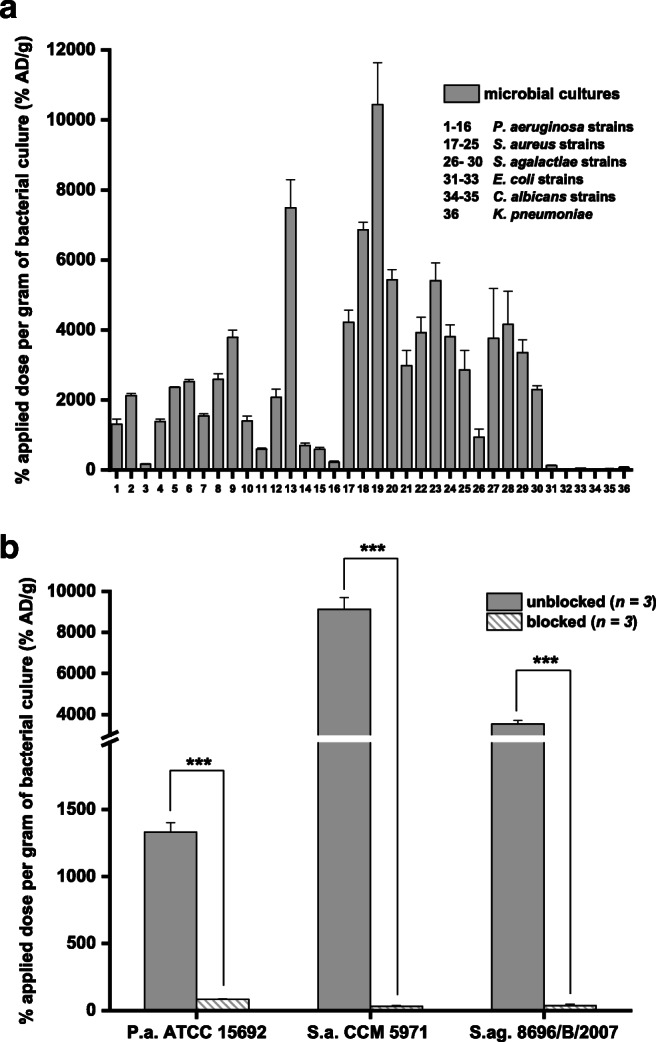
In vitro uptake of [^68^Ga]Ga-DFO-B in different microbial cultures 45 min after incubation (**a**) (1 = *P. aeruginosa* ATCC 15692, 2 = *P. aeruginosa* ATCC 10145, 3 = *P. aeruginosa* ATCC 9027, 4 = *P. aeruginosa* ATCC 27853, 5 = *P. aeruginosa* NCTC 10662, 6 = *P. aeruginosa* NCTC 12903, 7 = *P. aeruginosa* NCTC 12951, 8 = *P. aeruginosa* NCTC 13437, 9 = *P. aeruginosa* NCTC 6749, 10 = *P. aeruginosa* 3131, 11 = *P. aeruginosa* 3137, 12 = *P. aeruginosa* 3138, 13 = *P. aeruginosa* 1019, 14 = *P. aeruginosa* 10,321/B/2017, 15 = *P. aeruginosa* 9564/B/2017, 16 = *P. aeruginosa* 181–12/CF/2017, 17 = *S. aureus* 230, 18 = *S. aureus* CCM 5757, 19 = *S. aureus* CCM 5971, 20 = *S. aureus* CCM 5972, 21 = *S. aureus* CCM 5973, 22 = *S. aureus* CCM 7058, 23 = *S. aureus* CCM 7109, 24 = *S. aureus* CCM 7110, 25 = *S. aureus* CCM 7114, 26 = *S. agalactiae* 52/CF/2013, 27 = *S. agalactiae* 7832/B/2007, 28 = *S. agalactiae* 8696/B/2007, 29 = *S. agalactiae* 8793/B/2007, 30 = *S. agalactiae* 9929/B/2007, 31 = *E. coli* ATCC 10536, 32 = *E. coli* 71/CF/2020, 33 = *E. coli* 10/CRC/2014, 34 = *C. albicans* 145/NS, 35 = *C. albicans* 1265/IDE/2007, 36 = *K. pneumoniae* 5044/A/2020). In vitro uptake of [^68^Ga]Ga-DFO-B in selected strains of *P. aeruginosa*, *S. aureus* and *S. agalactiae* after 45-min incubation unblocked and blocked with excess of iron-DFO-B; ***P < 0.005 (**b**)

In vitro uptake of [^68^Ga]Ga-DFO-B was studied in various bacterial cultures. The highest uptake was observed in several strains of *P. aeruginosa*, *S. aureus* and *Streptococcus agalactiae*, while different strains of *E. coli*, *Candida albicans* and *K. pneumoniae* did not show any significant accumulation of [^68^Ga]Ga-DFO-B at 45 min after incubation (Fig. 2a). The high in vitro uptake of [^68^Ga]Ga-DFO-B in selected strains of *P. aeruginosa*, *S. aureus* and *S. agalactiae* could be blocked (Fig. 2b) with an excess of cold iron-DFO-B. Chemically, it is a complex. Similarly as e.g. [68Ga]Ga-DFO-B. indicating a specific uptake mechanism (*P* < 0.005).

### Biodistribution in healthy mice

**Fig. 3:**
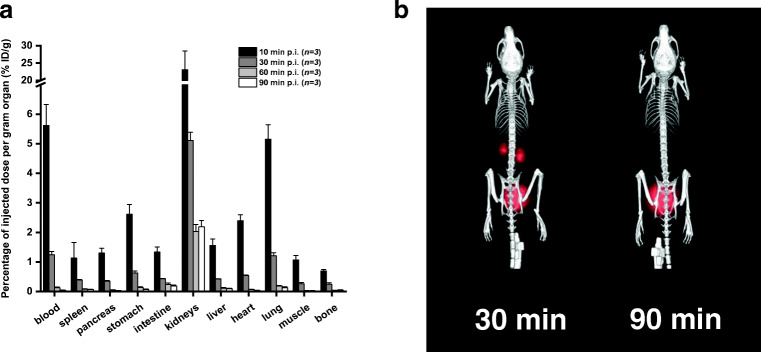
Ex vivo biodistribution of [^68^Ga]Ga-DFO-B in healthy Balb/c mice 10-, 30-, 60- and 90-min p.i. (**a**). Static PET/CT imaging (3D volume rendered images) of [^68^Ga]Ga-DFO-B in healthy Balb/c mice 30 and 90 min after injection (**b**)

The ex vivo biodistribution data as well as PET/CT imaging acquired from experiments with healthy Balb/c mice displayed rapid excretion of [^68^Ga]Ga-DFO-B via the renal system (23.1 ± 5.44 %ID/g 10-min p.i. versus 2.19 ± 0.21 %ID/g 90-min p.i.) and showed minimal retention in blood and other organs, even at short time (90 min) after injection (Fig. 3). Highest activity concentration in the organs at late time points (90-min p.i.) was found for kidneys (2.19 ± 0.21 %ID/g) and intestines (0.18 ± 0.04 %ID/g). The ex vivo biodistribution data were consistent with the results of PET/CT imaging 30- and 90-min p.i..

### Dosimetric calculations

Results from dosimetric calculations are presented in Supplementary Table S2. As expected from the rapid renal excretion of [^68^Ga]Ga-DFO-B, the organ receiving the highest adsorbed doses with 0.187 mGy/MBq is the urinary bladder wall, when no voiding is taken into account, considering a 1-h voiding interval this value decreases to 0.076 mGy/MBq. All other organs receive a dose of < 0.02 mGy/MBq independent on the voiding interval. The estimated human effective dose was 0.020 mSv/MBq total and 0.014 mSv/MBq with a bladder voiding interval of 1 h.

### PET/CT imaging and ex vivo biodistribution in animal infection models

**Fig. 4:**
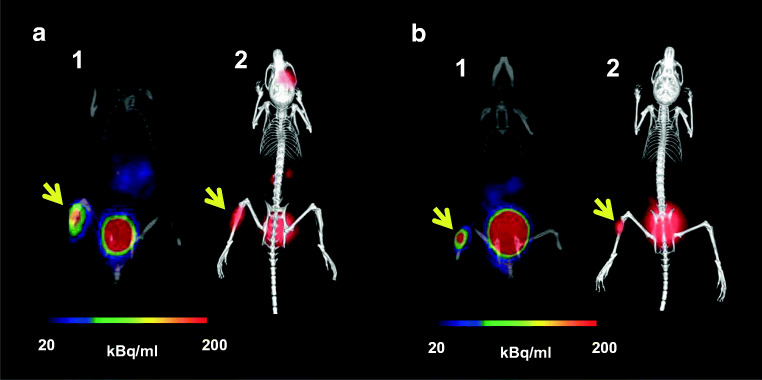
Static PET/CT imaging (coronal slices (1) and 3D volume rendered images (2)) of [^68^Ga]Ga-DFO-B in *P. aeruginosa* infected (**a**) and *S. aureus* infected (**b**) Balb/c mice 45 min after injection

**Fig. 5:**
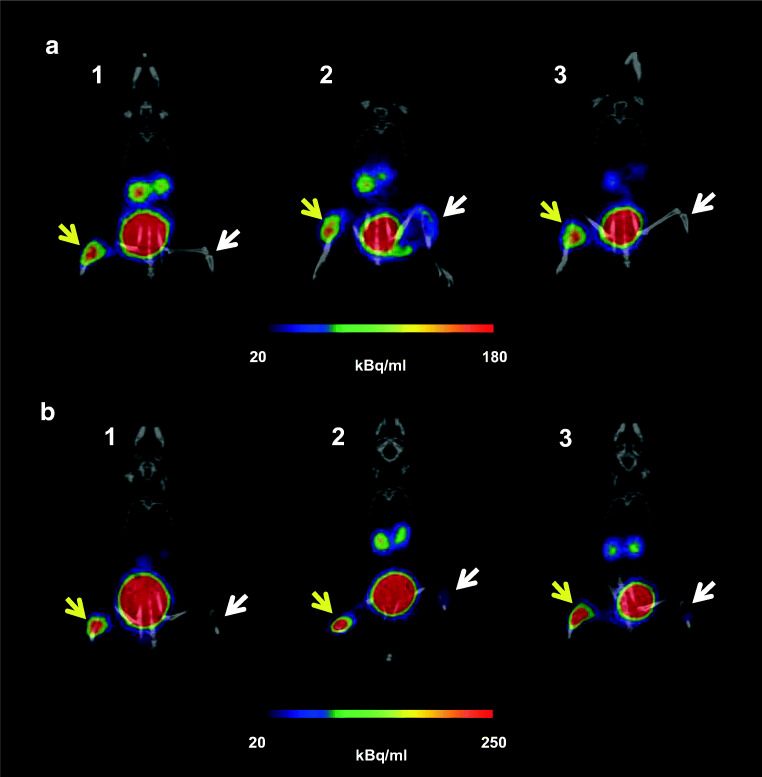
PET/CT images (coronal slices) of acute murine myositis 5 h after inoculation and 45 min after injection of [^68^Ga]Ga-DFO-B (**a**) *P. aeruginosa* versus *P. aeruginosa* heat-killed (1), *P. aeruginosa* versus sterile inflammation (2), *P. aeruginosa* versus *E. coli* (3); (**b**) *S. aureus* versus *S. aureus* heat-killed (1), *S. aureus* versus sterile inflammation (2), *S. aureus* versus *E. coli* (3); yellow arrow indicates *P. aeruginosa* or *S. aureus* infection, while white arrow pointing at *P. aeruginosa* or *S. aureus* heat-killed, sterile inflammation or *E. coli* infection)

**Fig. 6:**
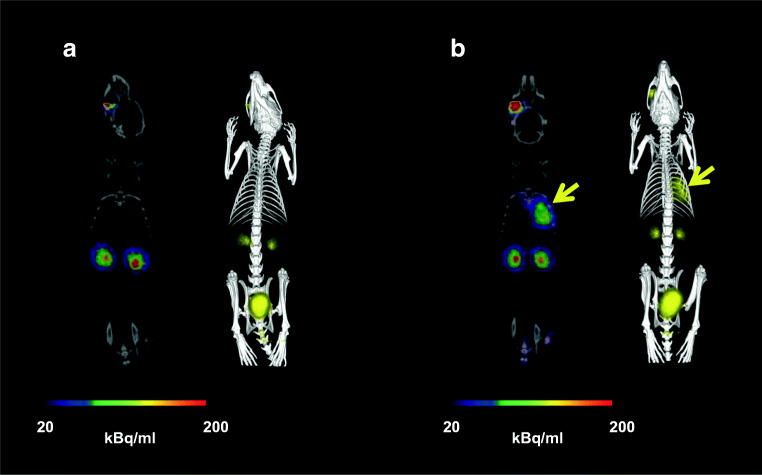
Static PET/CT imaging (coronal slices and 3D volume rendered images) of [^68^Ga]Ga-DFO-B in non-infected (**a**) and pulmonary infected (**b**) Lewis rats 45 min after injection; yellow arrow indicates *P. aeruginosa* infection

PET/CT images of acute murine myositis displayed specific accumulation of [^68^Ga]Ga-DFO-B in both *P. aeruginosa* and *S. aureus* infections (Fig. 4). [^68^Ga]Ga-DFO-B was taken up by live bacteria (both *P. aeruginosa* and *S. aureus*) injected in the left hind limbs of mice, while the right hind limbs treated with heat-killed bacteria, turpentine oil or live *E. coli* did not show any significant radioactive signal (Fig. 5). PET/CT imaging in the rat respiratory *P. aeruginosa* infection model showed focal accumulation of [^68^Ga]Ga-DFO-B in the lung (Fig. 6). No uptake in the lung region was detected in non-infected rats in which the only visible organs were the kidneys and bladder.

**Fig. 7:**
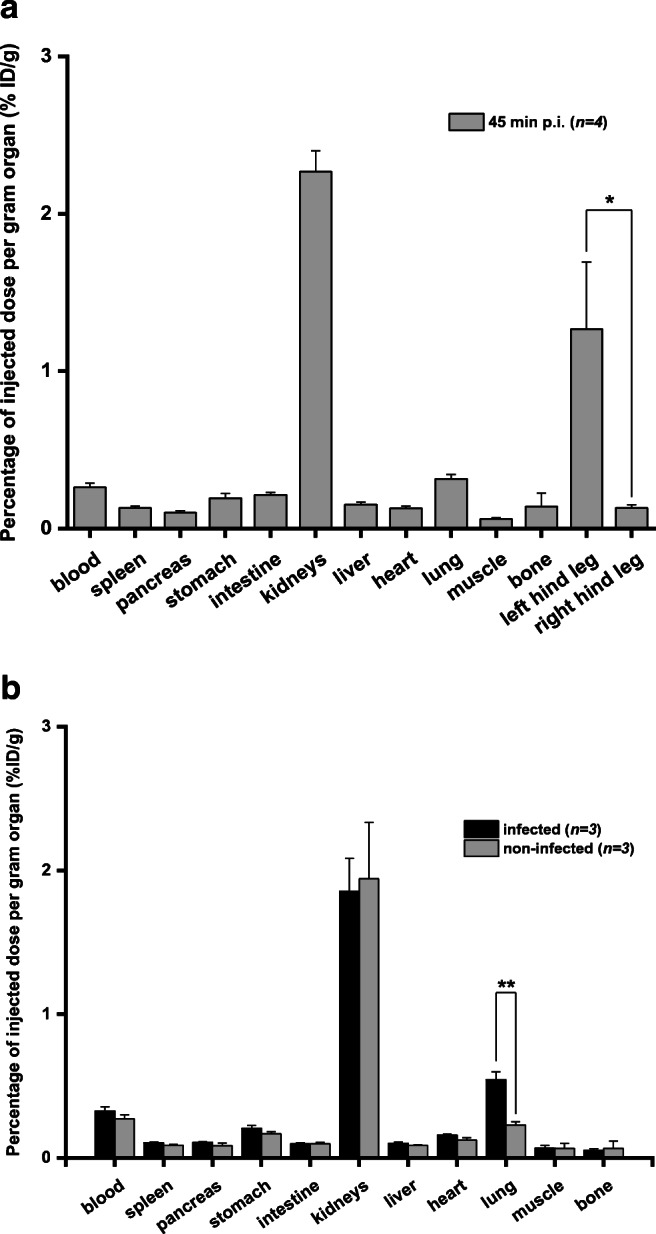
Ex vivo biodistribution of [^68^Ga]Ga-DFO-B in murine *S. aureus* myositis model; *P < 0.05 (**a**) and in intratracheally *P. aeruginosa* infected versus non-infected rats 45-min p.i.; **P < 0.01 (**b**)

Ex vivo biodistribution of [^68^Ga]Ga-DFO-B in acute *S. aureus* murine myositis revealed distinct difference between the uptake in the excised infected versus non-infected hind limbs (left hind leg: infected (1.27 ± 0.43 %ID/g) and right hind leg: non-infected (0.13 ± 0.02 %ID/g); *P* < 0.05). Similarly, [^68^Ga]Ga-DFO-B was tested in the rat respiratory *P. aeruginosa* infection model. Comparison of ex vivo biodistribution of intratracheally *P. aeruginosa* infected versus non-infected Lewis rats 45-min p.i. did not show any significant difference in organ uptake, except for lung uptake (0.54 ± 0.05 %ID/g in infected rats; 0.23 ± 0.02 %ID/g in non-infected rats; *P* < 0.01). Summary of ex vivo biodistribution studies in both *S. aureus* and *P. aeruginosa* infection models is depicted in Fig. 7.

## Discussion

To achieve bacterial specific accumulation with a radiotracer, an active transport mechanism, conceptually similar to [^18^F]fluorodeoxyglucose, resulting in sufficiently high accumulation of radionuclides in the infection, seems to be highly desirable for successful in vivo targeting. Recently, several small molecule pathogen-specific radiotracers have been reported, for review see [29], targeting differences in carbohydrate uptake and metabolism (e.g. [^18^F]fluorodeoxysorbitol, [^18^F]fluoromaltose and [^18^F]fluoromaltotriose), folate biosynthesis (e.g. para-[^11^C]aminobenzoic acid, para-[^18^F]fluoroaminobenzoic acid analogue and [^18^F]fluoropropyl-trimethoprim) or bacterial cell wall biosynthesis (e.g. radio-analogues of D-aminoacids labelled with ^11^C). The main advantages of using [^68^Ga]Ga-DFO-B as compared with these promising radiotracers are in particular (i) the use of ^68^Ga as a radiolabel enabling on-site preparation of the radiotracer in time of need, (ii) possibility of fast and reliable automated radiosynthesis without the requirement on cyclotron and (iii) application of well-established clinically used medication—Desferal®.

DFO-B has a long history not only as a medicinal iron chelator. DFO-B and its derivatives have been widely studied and used in the field of nuclear medicine as a bifunctional chelating agent enabling radiolabelling of wide range of biomolecules with different radionuclides e.g. ^67^Ga, ^111^In and more recently ^89^Zr for immuno-PET [30–38]. Early attempts were intended to use DFO-B to improve SPECT imaging of infection [39–41]. Recently, Ioppolo et al. [42] used DFO-B for labelling with ^67^Ga and included modifications to adjust pharmacokinetics for targeting *S. aureus* infections. They reported active uptake in bacterial cultures in vitro and selective uptake in infections in vivo, but used only *S. aureus* without any other control and failed to achieve suitable uptake in their models. In a previous study, we used ^68^Ga-labelled pyoverdine [26], a primary siderophore produced by *P. aeruginosa*. [^68^Ga]Ga-pyoverdine showed highly specific uptake in *P. aeruginosa*, which was not observed in other tested microorganisms including *S. aureus.* This confirmed the potential to use siderophores for bacterial imaging with high specificity targeting homologous siderophore transporters (STs), however, with the limitation of using a siderophore not approved as a drug with a limited field of application.

In this study, we have investigated, for the first time, the in vitro and in vivo behaviours of [^68^Ga]Ga-DFO-B and its potential to be used for the imaging of bacterial infections. [^68^Ga]Ga-DFO-B showed hydrophilic properties, rather low protein binding and high stability in human serum and PBS. In vitro assays in microbial cultures revealed high uptake of [^68^Ga]Ga-DFO-B by various microorganisms utilizing DFO-B as xenosiderophore. It is known that some microbes are capable of siderophore piracy. For instance, most bacteria are able to utilize iron bound to siderophores from different microorganisms, so-called xenosiderophores by heterologous STs. Although heterologous STs are less specific than homologous STs, which specifically recognize the Fe-siderophore produced by the same microorganism, xenosiderophore uptake is a common feature. In a natural competitive environments and considering the “battle for iron” between the invading pathogen and the host, it is frequent that bacteria express a variety of different homologous and heterologous STs enabling to acquire iron from the best available source. We have shown here that, although the basic mechanism of siderophore uptake is different between gram-negative and gram-positive bacteria [21], [^68^Ga]Ga-DFO-B can be taken up by representatives of both groups. Interestingly, the in vitro uptake of [^68^Ga]Ga-DFO-B was diverse not only among bacterial species, but also between individual bacterial strains. This could be related to the experimental setting (e.g. varying response to STs upregulation in the artificial setting in vitro) or reflect different genetically determined responses to “iron starvation”. However, the in vitro uptake in *P. aeruginosa*, *S. aureus* and *S. agalactiae* strains was always specifically detected. Considering the active STs mechanism, effective direct in vivo targeting of the infection seems achievable also in cases of lower in vitro uptake values. *P. aeruginosa* ATCC 15692 and *S. aureus* CCM 5971 as the representatives of gram-negative and gram-positive bacteria belonging to ESKAPE pathogens with confirmed high in vitro uptake of [^68^Ga]Ga-DFO-B were chosen for further in vivo experiments in animal infection models.

[^68^Ga]Ga-DFO-B displayed excellent pharmacokinetic properties in healthy mice and rats being in accordance with reports on other radiolabelled hydroxamate siderophores [42–44]. The estimated radiation dose of 0.02 mSv/MBq, even when considering no bladder voiding, indicates a very low radiation burden for patients, even below that of other well-established ^68^Ga-labelled radiopharmaceuticals. In acute murine myositis and respiratory rat models, [^68^Ga]Ga-DFO-B was distinctly accumulated at the sites of the infection, with high target to non-target ratios, indicating that despite the rapid pharmacokinetics and potential barriers such as biofilm or fibrosis, bacteria are sufficiently accessible for direct targeting in vivo by [^68^Ga]Ga-DFO-B*.* This is supported by the low molecular weight of the compound resulting in rapid diffusion combined with the active uptake mechanism via STs. The only other sites of the radioactive signal detected 45-min p.i. were the kidneys and urinary bladder. One of the major goals of bacteria-specific radiotracers is to distinguish microbial growth from the non-bacterial inflammation [29]. A murine model of myositis was used to determine the specificity of [^68^Ga]Ga-DFO-B for living bacteria in vivo. μPET/CT imaging using [^68^Ga]Ga-DFO-B demonstrated significant differences in the accumulation of the radiotracer into the site of live bacteria versus the site of heat-inactivated inoculation or sterile inflammation. Moreover, [^68^Ga]Ga-DFO-B accumulation, both in vitro and in vivo, by *E. coli* strains under study, was negligible showing the specificity of [^68^Ga]Ga-DFO-B for microorganism employing dedicated deferoxamine transporters [45].

Direct comparison between *P. aeruginosa* infected and non-infected rats as well as infected and non-infected hind limbs of mice inoculated with *S. aureus* displayed significant differences in radioactivity accumulation in infected versus healthy tissues. Ex vivo biodistribution studies were in full accordance with the data obtained by means of PET/CT imaging. Similar acute infection models have also been widely used for preclinical characterization of other promising radiopharmaceuticals for infection [29]. They may not always fully reflect the clinical situation of chronic infections or foreign body associated infections. This is a certain limitation also of this study. However, the crucial role of iron acquisition in infection in general (“battle for iron”) involving an upregulated active transport mechanisms [20–22] holds promise that also in this setting positive diagnosis can be achieved.

Overall, our investigations indicate that molecular imaging of bacterial infections with [^68^Ga]Ga-DFO-B is possible and highly promising. Even in bacteria which reportedly use predominantly heme iron acquisition during infection, such as *Pseudomonas* spp. [46], specific accumulation of radiolabelled DFO-B in vivo in two different animal models could be shown.

## Conclusion

We have shown that [^68^Ga]Ga-DFO-B can be used for the detection of different bacterial infections. The high and specific uptake of [^68^Ga]Ga-DFO-B by *P. aeruginosa* and *S. aureus* was confirmed both in vitro and in vivo, proving the potential of DFO-B for specific imaging of bacterial infections. Biodistribution studies in healthy animals showed excellent pharmacokinetics of [^68^Ga]Ga-DFO-B with rapid renal excretion and no accumulation in any other studied organ. Animal infection models displayed significant focal uptake of [^68^Ga]Ga-DFO-B in the infection sites. As DFO-B is accepted in clinic for many years and licenced generators for obtaining ^68^Ga in pharmaceutical grade have become available, we believe that [^68^Ga]Ga-DFO-B has a great potential for smooth clinical translation.

## Electronic supplementary material

ESM 1 (PDF 304 kb)
