# Supplementary material for: 68Ga-labelled desferrioxamine-B for bacterial infection imaging
Source: Eur J Nucl Med Mol Imaging. 2020 Jul 30;48(2):372–82. doi: 10.1007/s00259-020-04948-y (PMC7835195; doi:10.1007/s00259-020-04948-y)
Supplement: Supplementary file 1 — (PDF 304 kb) [file 259_2020_4948_MOESM1_ESM.pdf]

^{68}Ga-labelled desferrioxamine-B for bacterial infection imaging

European Journal of Nuclear Medicine and Molecular Imaging

Milos Petrik, Eva Umlaufova, Vladislav Raclavsky, Andrea Palyzova, Vladimir Havlicek, Joachim Pfister, Christian Mair, Zbynek Novy, Miroslav Popper, Marian Hajduch and Clemens Decristoforo

Corresponding authors:

Milos Petrik

Institute of Molecular and Translational Medicine, Hnevotinska 5, CZ-77900 Olomouc, Czech Republic

Tel: +420585632126; Fax: +420585632180; Email: milos.petrik@upol.cz

https://orcid.org/0000-0003-1334-5916

Clemens Decristoforo

Clinical Department of Nuclear Medicine, Medical University Innsbruck, Anichstrasse 5, A-6020 Innsbruck, Austria

Tel: +4351250480951; Fax:+435125046780951; E-mail: Clemens.Decristoforo@i-med.ac.at

https://orcid.org/0000-0003-0566-4036

Supplementary Fig. S1 Representative radio-HPLC chromatogram of [^{68}Ga]Ga-DFO-B, in comparison UV-chromatogram of DFO-B-standard (Desferal®).

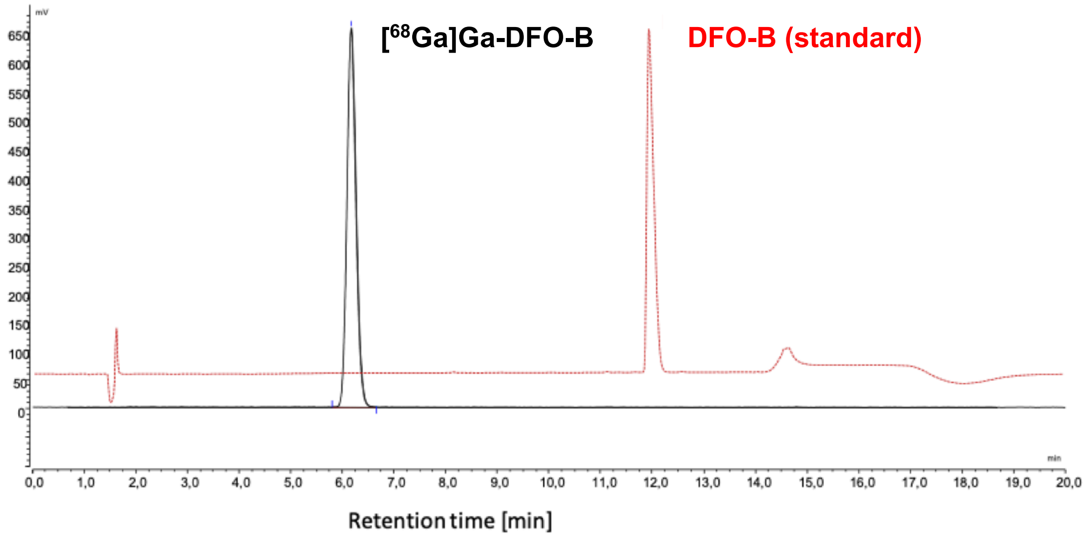

Supplementary Table S1 Microbial strains used in the study.

*Isolate No refers to the clinical isolates from the Department of Microbiology, Faculty of Medicine and Dentistry, Palacky University and University Hospital Olomouc, or public culture collections as indicated (ATCC, NCTC, CCM).

ATCC – American Type Culture Collection; NCTC – National Collection of Type Cultures; CCM – Czech Collection of Microorganisms; CF – cystic fibrosis.

**Species**	**Isolate No***	**Origin**
Candida albicans	145/NS	mouth wash of a lung cancer patient
Candida albicans	1265/IDE/2007	sputum of a CF patient
Escherichia coli	ATCC 10536	ATCC
Escherichia coli	71/CF/2020	sputum of a CF patient
Escherichia coli	10/CRC/2014	stool of a colorectal cancer patient
Klebsiella pneumonia	5044/A/2020	surgical wound of a urinary bladder cancer patient
Pseudomonas aeruginosa	ATCC 15692	ATCC
Pseudomonas aeruginosa	ATCC 10145	ATCC
Pseudomonas aeruginosa	ATCC 9027	ATCC
Pseudomonas aeruginosa	ATCC 27853	ATCC
Pseudomonas aeruginosa	NCTC 10662	NCTC
Pseudomonas aeruginosa	NCTC 12903	NCTC
Pseudomonas aeruginosa	NCTC 12951	NCTC
Pseudomonas aeruginosa	NCTC 13437	NCTC
Pseudomonas aeruginosa	NCTC 6749	NCTC
Pseudomonas aeruginosa	3131	nasal swab of a CF patient
Pseudomonas aeruginosa	3137	pharyngeal swab of a CF patient

Pseudomonas aeruginosa	3138	pharyngeal swab of a CF patient
Pseudomonas aeruginosa	1019	sputum of a CF patient
Pseudomonas aeruginosa	10321/B/2017	external ear channel of an otitis externa patient
Pseudomonas aeruginosa	9564/B/2017	external ear channel of an otitis externa patient
Pseudomonas aeruginosa	181-12/CF/2017	sputum of a CF patient
Staphylococcus aureus	230	sputum of a CF patient
Staphylococcus aureus	CCM 5757	CCM
Staphylococcus aureus	CCM 5971	CCM
Staphylococcus aureus	CCM 5972	CCM
Staphylococcus aureus	CCM 5973	CCM
Staphylococcus aureus	CCM 7058	CCM
Staphylococcus aureus	CCM 7109	CCM
Staphylococcus aureus	CCM 7110	CCM
Staphylococcus aureus	CCM 7114	CCM
Streptococcus agalactiae	52/CF/2013	sputum of a CF patient
Streptococcus agalactiae	7832/B/2007	traumatic wound
Streptococcus agalactiae	8696/B/2007	nasopharyngeal swab of a low-birth weight neonate
Streptococcus agalactiae	8793/B/2007	urinary tract infection
Streptococcus agalactiae	9929/B/2007	vagina of a breast cancer patient

Supplementary Table S2 Expected absorbed doses of [^{68}Ga]Ga-DFO-B in humans based on two considerations for bladder voiding intervals.

**Organ**	**Absorbed dose per injected activity [mGy/MBq]**	
	no voiding	*1h voiding interval*
Adrenals	0.013	0.013
Brain	0.013	0.013
Breasts	0.012	0.012
Gallbladder Wall	0.013	0.013
LLI Wall	0.015	0.014
Small Intestine	0.015	0.015
Stomach Wall	0.013	0.013
ULI Wall	0.014	0.014
Heart Wall	0.006	0.005
Kidneys	0.017	0.017
Liver	0.004	0.004
Lung	0.006	0.006
Muscle	0.005	0.004
Pancreas	0.005	0.005

Red Marrow	0.010	0.010
Osteogenic Cells	0.018	0.018
Skin	0.011	0.011
Spleen	0.004	0.004
Testes	0.014	0.013
Thymus	0.012	0.012
Thyroid	0.013	0.013
Urinary Bladder Wall	0.187	0.076
Total Body	0.013	0.012
Effective Dose [mSv/MBq]	0.020	0.014
